# Progress of Research on the Physiology and Molecular Regulation of Sorghum Growth under Salt Stress by Gibberellin

**DOI:** 10.3390/ijms24076777

**Published:** 2023-04-05

**Authors:** Jiao Liu, Yanqing Wu, Guichun Dong, Guanglong Zhu, Guisheng Zhou

**Affiliations:** 1 Joint International Research Laboratory of Agriculture and Agri-Product Safety, the Ministry of Education of China, Yangzhou University, Yangzhou 225009, China; 2 Jiangsu Provincial Key Laboratory of Crop Genetics and Physiology, Yangzhou University, Yangzhou 225009, China

**Keywords:** gibberellin, salt stress, sorghum, morphological indicators, physiological traits

## Abstract

Plant growth often encounters diverse abiotic stresses. As a global resource-based ecological problem, salinity is widely distributed and one of the major abiotic stresses affecting crop yields worldwide. Sorghum, a cereal crop with medium salt tolerance and great value for the development and utilization of salted soils, is an important source of food, brewing, energy, and forage production. However, in soils with high salt concentrations, sorghum experiences low emergence and suppressed metabolism. It has been demonstrated that the effects of salt stress on germination and seedling growth can be effectively mitigated to a certain extent by the exogenous amendment of hormonal gibberellin (GA). At present, most of the studies on sorghum salt tolerance at home and abroad focus on morphological and physiological levels, including the transcriptome analysis of the exogenous hormone on sorghum salt stress tolerance, the salt tolerance metabolism pathway, and the mining of key salt tolerance regulation genes. The high-throughput sequencing technology is increasingly widely used in the study of crop resistance, which is of great significance to the study of plant resistance gene excavation and mechanism. In this study, we aimed to review the effects of the exogenous hormone GA on leaf morphological traits of sorghum seedlings and further analyze the physiological response of sorghum seedling leaves and the regulation of sorghum growth and development. This review not only focuses on the role of GA but also explores the signal transduction pathways of GA and the performance of their responsive genes under salt stress, thus helping to further clarify the mechanism of regulating growth and production under salt stress. This will serve as a reference for the molecular discovery of key genes related to salt stress and the development of new sorghum varieties.

## 1. Introduction

Soil salinization is a widespread ecological problem. According to statistics, saline soils cover an area of about one billion hm^2^, accounting for approximately 10% of the global land area. Of the salt-affected land, 7700 hm^2^ is secondary saline soil, and 58% occurs in irrigated agricultural areas [1]. High soil salinity prevents crop roots from efficiently absorbing water and nutrients, which damages cells, organs, and tissues, slows metabolism, and inhibits growth, resulting in decreased crop yield and quality. In severe conditions, crop plants cannot survive and have reasonable productivity [2].

Sorghum (*Sorghum bicolor* L.), a C4 plant of the family *Gramineae*, is an important source of food, fodder, and bioenergy production. It is one of the world’s most important crops and is most widely cultivated in Asia, Africa, and North America. According to the FAO, the planting area of sorghum is about 4.379 × 10^7^ hm^2^ all over the world. In China only, the planting area is 5.63 × 10^5^ hm^2^, mainly distributed in northeast, north, and northwest China [3]. Sorghum has excellent stress resistance characteristics such as drought resistance, flood tolerance, salinity tolerance, and strong soil adaptability. In China, it is mainly used for food, forage, and energy production. It has made a great contribution to ensuring food security and developing animal husbandry. Compared with other crops, sorghum has the following advantages:

(1) More stress tolerance. Compared to maize, it is more tolerant to drought, flood, salinity stresses, etc. [4]. More important, sorghum can generally be grown in barren land such as in saline lands. This can set aside more arable land for food crops, such as *rice*, *maize*, *wheat*, etc. Furthermore, the saline soil was significantly improved after planting sorghum [5].

(2) Highly regeneration capacity. As forage sorghum, it can regenerate after harvest and grows quickly, allowing it to be harvested twice or three times in a single growing season [6]. The regeneration capacity makes sorghum seed once and repeatedly harvest, which saves labor and cost significantly.

(3) High-yielding and high-quality. As the forage sorghum plant is tall and stout, the stalks are juicy, and the stems and leaves are luxuriant. It can produce 52.5–75 tons/ha of silage, with the silage yield much higher than that of forage maize. For sorghum, 4500–7500 kg/ha of seeds can be harvested, and the nutritional composition is equivalent to or better than that of forage maize.

(4) Good hybridization. The hybrid sorghum has stronger growth potential than its parents, with luxuriant stems and leaves and well-developed root systems. The root cells have higher osmotic pressure, meaning they can absorb water from drier and earlier or saline soils to meet growth needs.

(5) Implement grass-fed crop rotation. China’s grassland wasteland area is about 26 million hectares. These grassland resources can be used to plant forage sorghum, and the implementation of grass-fed crop rotation improves yields, increases economic benefits, and reduces the competition between food production and other land uses. Compared with other field crops such as rice and wheat, sorghum is moderately tolerant to some abiotic stresses, including drought, flooding, soil barrenness, and salinity. In some soils with high salinity, however, sorghum faces practical problems such as difficulties with germination and emergence, delayed seedling growth, and lower yield potential.

However, sorghum experiences low emergence and suppressed metabolism in soils with high salt concentrations. It has been seen that the effect of salt on germination and seedling growth can be effectively mitigated to a certain range by the exogenous amendment of hormonal gibberellin. Gibberellins (GA_3_ is the major type) [7] are important plant hormones that are widely used to regulate plant growth during the whole life cycle of crop plants. They allow for plant growth and development by promoting cell increase and elongation, e.g., growth transformation of seedlings, nutritional development, fruit ripening, and so on [8,9]. In recent years, research on sorghum has developed rapidly and led to advances in the hormones involved in sorghum growth, physiology, and molecular mechanisms under salt stress.

In this paper, we have aimed to provide a comprehensive review of the effect of advances in the understanding of the roles of gibberellins on the growth, physiology, and molecular mechanisms of sorghum under salt stress, intending to better understand the roles of gibberellins in improving the yield and quality of sorghum grown in saline soils.

## 2. Effects of Salt Stress on Germination and Emergence of Sorghum

Salt stress is a key factor limiting sorghum germination in saline conditions. Previous studies have indicated that low concentrations (<50 mM) of NaCl promoted germination, while high concentrations (>100 mM) significantly inhibited germination [10,11]. At low NaCl concentrations, Na^+^ enters the seeds and increases the osmotic potential to promote water uptake and activate the activities of some enzymes involved in germination [12]. High NaCl concentrations, however, inhibit germination due to severe osmotic stress, which prevents the seeds from completing swelling and water uptake, coupled with the ionic toxicity caused by Na^+^ and Cl^−^ [13]. There are many other reasons why salt stress inhibits sorghum seed germination, and it is hypothesized that salt may alter the structure of proteins through a variety of mechanisms, thereby reducing the reserve capacity of seeds during germination, as well as enhancing seed respiration and accelerating nutrient consumption, to the detriment of seed germination and seedling emergence.

The root system is the major organ of sorghum for water and nutrient uptake. Under salt stress, the morphological construction of the sorghum root system, total root length, root surface area, root volume, and root tip number is significantly inhibited. Salt stress not only affects the morphological architecture of the root system but also causes changes in the anatomical structure of the root system, such as the root crown, elongation, and maturation. In addition, salt stress can directly affect the vigor of sorghum roots and limit the uptake and conductance of water and nutrients [14]. High salt concentrations increase the relative salt damage rate of the root system, leading to the disruption of intracellular ionic balance and the accumulation of a large amount of Na^+^ in the cell through external and intraplant electrochemical gradients, which in turn inhibit the uptake of Mg^2+^, Ca^2+^, and K^+^ ions and thus trigger monosilane toxicity [15,16].

Salt stress affects the external morphology of the plant, most visually felt in plant height [17]. The effect of salt stress on sorghum plant height varies between fertility stages, with plant height becoming lower as salt stress concentrations increase. The seedling stage is the most sensitive to salt stress, and salt stress at the gestation stage has the greatest effect on plant height, followed by the nodulation, tillering, and tasseling stages, with no significant effect on plant height at the grain filling and maturity stages. Salt stress also shortens the development of main sorghum stems, causes earlier onset of reproductive structures, shortens flowering time, and accelerates plant maturity [18].

**Figure 1:**
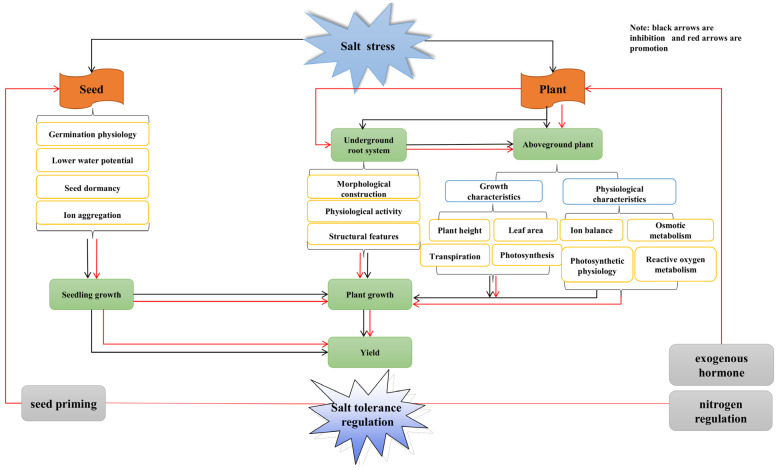
Effect of salt stress on sorghum growth physiology and salt tolerance regulation network figure.

Salt stress is one of the major abiotic stresses that inhibit plant growth and development. Morphological changes and growth indicators are the combined response of plants to salt stress and are important indicators of plant salt tolerance [19]. The response of plants to environmental changes is ultimately reflected in their external manifestations, with salt stress significantly affecting the growth of sorghum seeds and seedling plants. Previous studies have shown that with increasing NaCl concentrations, sorghum seed embryos are restricted in their ability to absorb water and even fail to germinate; the uptake of soil water and inorganic nutrients by the lower part of the ground is significantly reduced, and organic matter accumulation above ground is significantly decreased. This indicates that sorghum growth is more strongly inhibited under salt stress (Figure 1).

## 3. Effects of Gibberellins on Germination and Emergence of Sorghum under Salt Stress

Gibberellins (GAs) are plant endogenous growth regulators that promote cell division and germination and break bud dormancy in plants under abiotic stress [20]. Exogenous GA amendment at appropriate levels can promote the germination and emergence of sorghum under salt stress. An et al. found that 144 μM and 288 μM gibberellic acid successfully promoted water uptake and the germination of sorghum seeds [21,22]. The mechanism behind this is that GA_3_, at suitable levels, can promote the water uptake of sorghum seeds, increase the synthesis and activities of a variety of enzymes associated with germination, and a GAs, however, inhibit sorghum germination, emergence, and seedling growth [23,24]. In a previous study, 576 μM GA_3_ inhibited the water uptake of sorghum seeds and consequently reduced germination, emergence, and early seedling growth [9]. High concentrations of gibberellic acids can also cause osmotic stress, interfere with the normal metabolism of proteins and nucleic acids, inhibit cell differentiation and development, and thereby slow down plant growth [25].

It has been demonstrated that GA_3_ is synthesized at the root tip or leaf tip, and root growth depends on GA_3_ regulation of cells in the apical root meristem and elongation zone, while excessive salt induces an increase in ABA, which inhibits root apical growth hormone and GA_3_ synthesis and causes accumulation of DELLA protein in elongation zone cells and inhibits root growth and development by limiting cell proliferation and elongation [26,27]. GA_3_ at suitable levels can alleviate the stress of salt on the root system of sorghum plants. On the one hand, foreign amendment of GAs can alleviate cell wall thickening and elevate the cellulose and hemicellulose content of root cells caused by salt stress, allowing the rapid entry of water into root cells. On the other hand, the amendment of exogenous GAs can also change the dynamic balance of endogenous hormones in cells and combine with various receptors to induce the expression of related ions and enzymes. GA participates in the antioxidant system, promotes root development and alleviates damage induced by stress. Furthermore, Transcription factors in plant cells are also very important elements of plant responses to salt stress. Two types of transcription factors and genes have been identified to be involved in the salt stress response. One group is comprised of the transcription factors *NAC*, *AP2*, *MYB* and *WRKY*, which are mainly involved in salt stress responses and regulate root growth and development [28,29]. The other group is comprised of *NHX* and *NHA*, which regulate ion metabolism and transport, as well as peroxidase (POD) and catalase (CAT), which are related to the response to salt stress and ROS scavenging [30,31]. These proteins are all involved in the response of plant roots to salt stress. However, the response of these proteins needs to be further investigated in sorghum cultivars under salt stress.

GA_3_ is commonly used to regulate the growth of sorghum seedlings and has a growth-promoting effect on plant growth under salt stress [32]. It was found that GA_3_ treatment in low salt environments reduced leaf stomatal resistance, accelerated transpiration rate and increased water utilization, but exogenous GA_3_ had no significant effect in reducing plant growth inhibition at high salt concentrations. The reason for this may be that GA_3_-dependent reduction in stomatal resistance and increase in stomatal conductance allows transport and accumulation of toxic ions from roots to stems and leaves with transpiration under salt ribbing stress, increasing the toxic effect of toxic ions on sorghum cells.

GA_3_ initiation under salt stress not only promotes plant seed germination but also changes the effect on seedling growth and development in different plants. However, it significantly increased the sorghum leaves and yield of sorghum under salt stress.

It has demonstrated that GA_3_ played a positive role in alleviating sorghum salt stress, which provides important clues to understanding the defense mechanism of sorghum salt stress.

Figure 1. Salinity stress mainly affects seed germination and plant growth in sorghum. This reduces germination rates or results in dormant seeds that do not germinate, making seedling emergence difficult and inhibiting seedling growth, ultimately leading to yield reduction by affecting plant growth. The effects on plant growth are divided into those on the morphology and physiology of the below-ground root system and those on the growth and physiological metabolism of the above-ground plant. Salinity stress affects plant growth and yield by affecting the morphological structure, structural characteristics and physiological activity of the lower ground root system, which in turn affects water and nutrient uptake and utilization; it also affects plant growth and yield by reducing the accumulation and transport of dry matter through reduced leaf expansion, transpiration and photosynthetic characteristics of the plant population, and by affecting physiological processes such as ion balance, osmotic metabolism, photosynthetic physiology and reactive oxygen metabolism. The plant’s growth and yield are affected by physiological processes such as ion balance, osmotic metabolism, photosynthetic physiology and active oxygen metabolism.

## 4. Effect of Salt Stress on the Physiology of Sorghum

### 4.1. Regulation of Photosynthesis of Sorghum under Salt Stress

Photosynthesis is the material basis for all plant life activities, and salt stress can lead to a reduction in photosynthesis in plants (Figure 1). Photosynthesis in sorghum mainly occurs in the leaves. The organelle most closely associated with photosynthesis is the chloroplast, which is more sensitive to salt stress. Salt stress disrupts the structure of chloroplasts, reducing the chlorophyll content in the plant and preventing photosynthesis from taking place. The main effect is the inhibition of CO_2_ diffusion into the chloroplast. The results showed that the content of light and pigments in sorghum leaves decreased as the duration of stress gradually increased on the sorghum leaf. Salt stress can significantly reduce the content of photosynthetic pigments in leaves [33]. The expression of chlorophyll synthesis genes is downregulated, along with nuclear photosynthetic genes, through reverse signal transduction from the plastid to the nucleus. This, in turn, reveals the extent of salt stress damage to sorghum seedlings.

### 4.2. Regulation of the Antioxidant System of Sorghum under Salt Stress

Superoxide dismutase (SOD) and POD activities are widely used as indicators of stress resistance, and their activity levels can be used to evaluate plant tolerance to stress. CAT, an enzyme of the membrane protection system, can scavenge hydrogen peroxide in plants during stress, reduce the formation of oxygen radicals, maintain the balance of reactive oxygen metabolism in the plants, delay or hinder the destruction of cell structure, and keep tissues alive [34]. The redox system of salt-tolerant and salt-sensitive sorghum was studied by Sun et al. The SOD, CAT, POD and APX activities in sorghum seedlings under salt stress also increased with increasing salt concentration, especially in salt-tolerant varieties. The sorghum seedlings were also found to be more resistant to salt stress by enhancing the activity of antioxidant enzymes and thus avoiding the excessive accumulation of ROS and lipid peroxidation in the cell membrane [35]. Thus, the antioxidant-reduction system of sorghum has the ability to scavenge ROS produced by salt-induced oxidative stress, making salt-tolerant sorghum tolerant under saline conditions. On the other hand, the antioxidant reduction system of salt-sensitive sorghum varieties is not sufficient to undo salt-induced oxidative damage, resulting in fine membrane leakage, cell rupture, and, ultimately, plant death.

### 4.3. Regulation of Osmotic Substances and Ion Balance of Sorghum under Salt Stress

Generally, plants are injured by osmotic stress in parallel with salt stress, resulting in physiological drought [36]. Meanwhile, plants mainly mitigate the damage caused by salt stress through osmoregulation. One type is organic osmoregulatory substances such as synthetic proline, carbohydrates, and proteins. The other is dependent on inorganic osmoregulatory substances, such as Na^+^ and K^+^ ions [37,38].

The accumulation of proline (Pro) is thought to be an important mechanism for osmoregulation in plants under a variety of abiotic stresses [39]. Under salt stress, plant tissues accumulate Pro to mitigate the toxic effects of excess ammonia on the organism, keep the plasma membrane more intact for scavenging free radicals, and regulate osmotic pressure in the plant to prevent the plasma membrane from becoming permeable. In addition, it was found that the soluble protein, soluble sugar, and betaine contents of sorghum leaves are elevated and organic acids accumulate in the roots when exposed to salt stress [40]. Compared to non-salt-tolerant crops such as *rice* [41] and *maize* [42], osmoregulatory substances are substantially reduced, suggesting that organic osmotic substances in sorghum will collectively resist plant damage from salt stress and that non-salt-tolerant varieties will lose some of their protection from organic osmotic substances [43,44].

Potassium (K), one of the numerous nutrients in plants that ensure normal metabolism and growth, and development, has physiological functions such as the regulation of ionic balance, osmotic pressure, cell expansion, and photosynthesis [45,46]. The physicochemical properties of Na^+^ and K^+^ are similar in crop plants [47]. Na^+^ competes for the major binding sites of K^+^ in key cytoplasmic metabolic processes, leading to K^+^ loss [48]. Salinity stress increased Na^+^ uptake and decreased Ca^2+^ uptake [49,50], with small changes in Mg^2+^ content. Under salt stress, the content of Na^+^ in the root system was significantly higher than that in the stem and leaves, while the content of K^+^, Ca^2+^, and Mg^2+^ had the opposite trend. This indicates that Na^+^ and Ca^2+^ ions are the keys to the osmoregulation of crop plants under salt stress [51].

## 5. Effect of Gibberellins on Physiological Properties of Sorghum Plants under Salt Stress

### 5.1. Regulation of Photosynthesis in Sorghum under Salt Stress by Gibberellins

**Figure 2:**
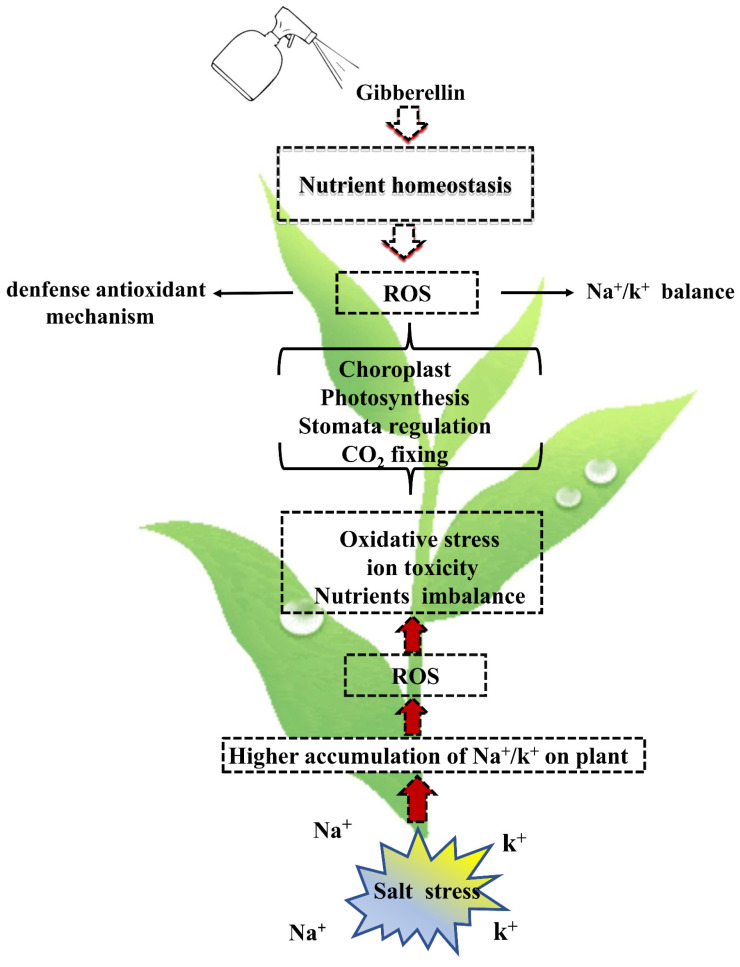
Functions of GAs in the regulation of photosynthesis under salt stress. In the absence of GA, salt stress results in an imbalance of Na^+^/K^+^ homeostasis, which leads to the production of ROS. This salinity-induced ROS production, in turn, exerts oxidative stress on plants, resulting in stomatal closure and reduced activity of CO_2_-fixing enzymes, resulting in a decrease in photosynthesis. In the presence of GA, Na^+^/K^+^ homeostasis, and nutrient homeostasis are maintained, and the antioxidant defense mechanism is activated, which limits ROS production, thereby preventing ROS-induced oxidative stress. In the absence of oxidative stress, the rate of photosynthesis is maintained during salt stress.

Chlorophyll is the main pigment for plant photosynthesis. GA_3_ plays a vital role in improving chlorophyll synthesis, and plant growth, especially under stress conditions [52] (Figure 2). Guo et al. [53] showed that the chlorophyll content of gibberellin-treated sesbania plants was higher than the control. Similar results were found by Erbil, who reported that GA_3_ application caused a significant increase in chlorophyll content in salt-stressed groundnut plants (*Arachis hypogaea* L.) [54]. The increase in chlorophyll content might be because the application of exogenous GA reduced the enzyme activities involved in chlorophyll catabolism and alleviated the oxidative chlorophyll bleaching, thereby inhibiting chlorophyll catabolism [55]. It is hypothesized that it may be that GA_3_ mitigates the damage to chloroplasts by harmful ions by enhancing the stability of the cystoid membrane, thus promoting the synthesis of photosynthetic pigments in leaves [56], but the damage to plants by salt stress is irreversible and exogenous GA_3_ cannot bring plants to CK levels in the short term. This is consistent with the results of the above-mentioned scholars but not with the trend of changes in photosynthetic pigment content of seedling leaves under salt stress alleviated by GA_3_, as studied by Zhang et al. [57]. This may be caused by many differences, such as different test materials or different salt stress concentrations.

### 5.2. Regulation of Antioxidant System in Sorghum under Salt Stress by Gibberellins

Beninca et al. found that GA is able to restore the antioxidant enzyme activity reduced by salt stress [58]. Under salt stress, reactive oxygen species (ROS) can damage functional macromolecular structures such as intracellular proteins and unsaturated fatty acids, leading to cell membrane lipid peroxidation [59]. After GA application, the activity of SOD increases, acting as the first line of defense for scavenging ROS. POD is a highly active enzyme that is associated with respiration, photosynthesis, and the oxidation of growth hormones, and its activity changes continuously during seedling growth and development [60]. The POD activity of sorghum seedlings under salt stress decreases significantly, and exogenous GAs are able to significantly increase the activity of this enzyme and alleviate the toxic effects of salt stress [61]. In the meantime, CAT activity also increases to scavenge excess ROS in sorghum plants [62].

### 5.3. Regulation of Osmotic Stress and Ion Balance in Sorghum under Salt Stress by Gibberellins

The amendment of exogenous GA can reduce Na^+^ and Cl^−^ uptake and transport to the above-ground part of plants, promote selective uptake of K^+^ and Ca^2+^ ions, partially increase the K^+^/Na^2+^ ratio in plant seedlings, and thus improve the seedling’s salt resistance and adaptation to salt stress. Zhu [63] at ‘*Paulownia 1201*’ seedlings found increased K^+^/Na^+^, Ca^2+^/Na^+^ and Mg^2+^/Na^+^ values in both leaves and roots and greater in leaves than in roots. This may be due to the fact that a large amount of Na^+^ is trapped and segregated in the root system by high-affinity transport proteins of other elements (K, Ca, Mg, etc.), which promotes the release of K^+^, Ca^2+^ and Mg^2+^, thus reducing the Na^+^ content in the above-ground plant cells to improve the salt resistance of the plant. The side effects of GA_3_ spraying promote the selective transport of K^+^, Ca^2+^ and Mg^2+^ by seedlings, thus improving the ionic balance between organs and meeting plant nutrient needs, which is also a mechanism for plant resistance to salt stress. In addition, similar results were found in maize [64], sweet sorghum [16], spinach [65], and other plant studies where exogenous hormones were effective in alleviating ion toxicity.

## 6. The Molecular Mechanisms of Sorghum under Salt Stress

The response of plants to salt stress is a complex physiological process. When plants are exposed to salt stress, they first respond at the genetic level through transcriptional regulation, followed by the synthesis of RNA coding for proteins associated with salt stress, and then through the fine control of metabolite biosynthesis, which regulates plant metabolism and osmotic balance.

### 6.1. RNA Sequencing (RNA-seq) Transcriptome Analysis of Sorghum under Salt Stress

Transcriptomics is an important way to study the changes in expression regulation at the transcriptional level. It can be used to explore differences in gene expression levels between individual tissues and gene structure, thus helping to reveal the molecular mechanism regulating the occurrence of stress responses in crop plants.

The responses of sorghum to salt stress have been relatively well studied in terms of morpho-physiological and biochemical aspects, but studies on the molecular mechanisms of salt tolerance in sorghum under salt stress have not been well documented. Transcriptome sequencing analysis of salt-stressed sorghum seedlings can quickly screen out stress-responsive genes in sorghum, clarify related metabolic pathways, and help us to analyze the function and regulatory mechanism of differential genes in sorghum under salt stress [66].

The strength of salt tolerance in sorghum under salt stress is largely dependent on differential gene expressions in salt-tolerant materials. In some early transcriptome analyses of *Arabidopsis thaliana* [67], a model plant, salt stress resulted in hundreds of differentially expressed genes (*DEGs*). Abreha’s [17] transcriptome study of sorghum revealed that differentially expressed genes are functionally enriched in catalytic activities, transport activities, functional molecular regulators, and enzyme regulatory activities, mainly involved in cellular processes, metabolic processes, stimulatory responses, biological regulation, and the regulation of biological processes [68]. This indicates that sorghum has relatively abundant gene expression and regulatory activities to cope with salt stress [69]. Based on a Gene Ontology (GO) enrichment analysis, Wang et al. found that sorghum was significantly enriched for differential genes in salt stress regulation, with the highest number of differentially expressed genes in the comparison group and at 12 h and 24 h, the most expression of ploidy 1–3-fold, and a large difference in number from the other comparison groups, containing 62 upregulated expressed genes and 33 downregulated expressed genes.

Based on the differential expression of genes in the transcriptome of sorghum seedling leaves under salt stress, KEGG was found to provide a more intuitive picture of the metabolic pathways we are studying [70]. The metabolic expression pathways involved are divided into five branches, namely cellular processes, environmental information processing, genetic information processing, metabolism, and organic systems. In the 0 h and 12 h salt stress comparison groups, there were 50 differential genes in the metabolic pathways, with the most diverse pathways in Metabolism and Photosynthesis. The largest number of differentially expressed genes was 28, accounting for 12.61% of the total differentially expressed genes annotated. The next most abundant pathway was the Plant Hormone Single Transduction pathway, which contained 21 differentially expressed genes, accounting for 9.46% of the total differentially expressed genes [71]. The remaining genes were related to osmoregulation, membrane lipid metabolism, salt signaling, and other pathways [72].

#### 6.1.1. Related Pathways of Hormone Signaling

Phytohormones are key regulators of physiological processes in plants over their whole life cycle, in normal growing conditions and under stress. Metabolic processes and hormonal signaling cascades are appropriate targets for manipulations aiming to improve plants’ stress resistance. Various plant life activities are regulated by plant hormones [73]. Some studies have shown that [74] the specificity of hormone regulation in plants is not obvious, and usually, one hormone can regulate multiple physiological processes. It is also common for multiple hormones to regulate the same physiological process in concert [75]. The interactions between these hormones explain the damage caused by salt stress in sorghum.

(1) Ethylene exerts a dual effect on sorghum survival under salt stress, either positively or negatively. Ethylene receptors have been shown to interact with ABA production, thus delaying germination and inducing the survival of sorghum seeds.
(2) Cytokinins (CKs), along with auxin, are involved in the maintenance of both shoot and root meristems by stimulating stem cell division [76]. Contrasting data, however, were reported on the role of CKs in the salt stress response that supports both negative and positive effects [77]. Studies on the activity of CK receptors demonstrated an interplay with ABA in transducing abiotic stress signals [78], which in leaves interferes with ABA-induced stomatal closure [79].
(3) Auxin is mainly involved in the development and determining of the shape of the plant body by controlling cell division and developmental patterning. The action of auxin is strictly related to its concentration, and the direction of growth of an emerging organ is determined by auxin gradients. Changes in growth hormone concentration gradients have been shown to improve salt tolerance by stimulating sorghum growth and increased biomass.
(4) Jasmonate and salicylic acid have been reported to jointly study the tolerance of sorghum salt stress by interacting with other hormone pathways and reducing oxidative stress [80].
(5) GAs and brassinosteroids, implicated as well as auxin in cell division, have been reported to play a role in salt tolerance by helping in the recovery phase [81] or by interplaying with ROS [82] and other hormones [83].

**Figure 3:**
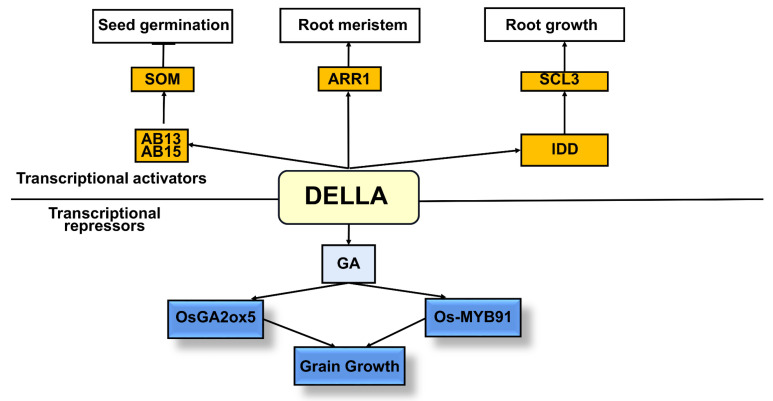
Molecular regulation mechanism of gibberellins in rice under salt stress.

GAs level manipulation seems a promising approach for obtaining new varieties with improved salt tolerance. Some studies have shown that salt stress also reduces enzyme activities, as well as hampering the nutritional balance in plants. A study found that OsCYP71D8L is a potential GA-deactivating protein that plays a significant role in balancing the growth process and stress responses and leads to enhanced tolerance to salt stress in rice [84]. In contrast, OsCYP71D8L belongs to the Delara family of proteins and is a major GA-negative regulator involved in environmental and hormonal signaling. In addition, overexpression of other GA catabolism-related genes in rice, such as *OsGA2ox5* [85] and *Os-MYB91* [86], and overexpression of *AtGA2ox7* [87] in *Arabidopsis* decreased growth and its tolerance to salt stress was enhanced. This result suggests that the reduction of GA signaling under salt stress conditions is directly associated with salt tolerance in plants. On the other hand, some papers have reported that the exogenous application of GA had a positive effect on salt stress tolerance in many crops. In this context, increased GA biosynthesis is one of the important mechanisms affecting salt stress tolerance in plants. At present, the research on the DELLA family protein in sorghum is still inadequate. However, overexpression of *OsGA2ox5* and *OsMYB91* in *rice* and *AtGA2ox7* in *Arabidopsis* and other GA catabolism-related genes can reduce growth and show an adverse reaction, with enhanced tolerance to salt stress (Figure 3).

**Figure 4:**
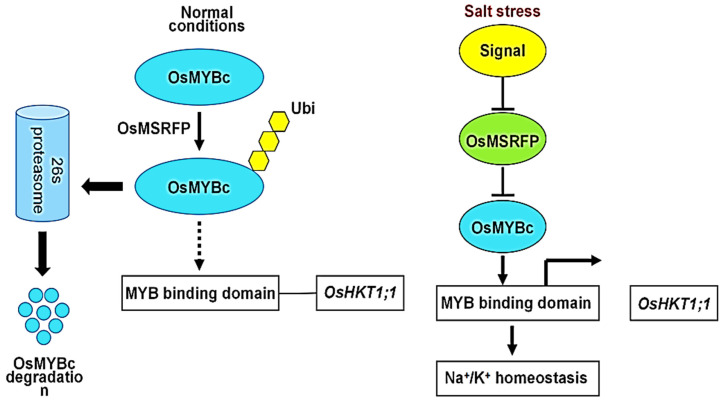
Molecular regulation mechanism of gibberellins in plants under salt stress.

Salt tolerance in sorghum is largely dependent on differences in gene expression in salt-tolerant materials. According to the latest sorghum RNA-seq data, salt stress induced the gene *Sb10g000920* [88] encoded in the roots of the salt-tolerant test material M-81E in sorghum, whereas no DEG has been associated with GA biosynthesis in the salt-sensitive material Roma. *Sb06g019600* encoding *CYP 724B1* was expressed downregulated in both leaves and roots of M-81E. This indicates that *Sb06g019600* encoding *CYP 724B1* is salt tolerant to sorghum. Most of the hormone-related genes in the study play a positive regulatory role, and qRT-PCR validation results indicate that they are able to respond uniquely to salt stress. Members of the transcription factor family, such as *bZIP*, *WRKY*, *AP2*/*EREBP*, *C2H2*, *bHLH*, *MYB* and *NAC*, have now been shown to be involved in the plant response to salt stress [89] (Figure 4). *Sb06g019600* encoding *CYP 724B1* in the sorghum study suggests that they are functional fragments in response to salt stress. Fang et al. [90] examined sorghum stem, leaf, root and shoot tissues and found that the expression of the *SbSBP15* gene in sorghum was significantly higher in stems than in other tissues and more delayed in response to salt stress. However, *SbSBP15* gene expression showed a rapid and significant inhibition under GA treatment, very similar to that of *CmSPL6*, *CmSPL9* and *CmSPL16* [91] in response to GA.

In conclusion, when sorghum is under salt stress, phytohormones can induce transcription factors, photosynthesis, and antioxidant activity in sorghum to alleviate stress. This is consistent with Guo et al.’s research. The changes in transcription factors, genetic material, antioxidant activity and plant hormone signal transduction constitute the mechanism and strategy of sweet sorghum seedlings to resist salt stress. The mechanism of hormone signal transduction in sorghum under salt stress is still unclear. It remains a great challenge that can be only resolved by moving from whole plant studies (employed by 95% of published papers) to more in-depth studies at the cellular level, using a modern range of biophysical and imaging techniques that allow quantification of the operation of key transport systems conferring plant ionic and oxidative homeostasis under stress conditions.

#### 6.1.2. Related Pathways of Photosynthesis

Photosynthesis is the main mode of energy production during plant growth and development [92]. Photosynthetic electron transport is the main step in photosynthesis; it is a process that converts light energy into chemical energy and provides ATP for subsequent carbon assimilation [93]. It has been shown that genes that are involved in chlorophyll synthesis, photosystem II (PSII), photosystem I (PSI), cytochrome, adenosine triphosphate (ATP) [94], synthase, and carbon fixation pathways play a key role in plant salt tolerance. A study on salt tolerance mechanisms in sorghum revealed that 84 differentially expressed genes (*DEGs*) are associated with photosynthesis in salt-tolerant crops after salt stress. During stomatal regulation and osmotic adjustment in sorghum in response to salinity [93], up to 51 genes are related to chlorophyll synthesis; most *DEGs* are related to chlorophyll, PS II, and cytochrome, and all the *DEGs* related to ATP synthase and carbon fixation are upregulated [95]. This suggests that salt stress can induce photosynthetic response genes in crops, and these genes related to photosynthetic electron transport may be activated by strong light energy conversion [96].

Homeostasis of essential elements such as N, P, K, S, and Ca is altered during salt stress, which in turn affects the photosynthetic efficiency of plants [97]. Additionally, salt-tolerant sweet sorghum can maintain a high sugar content in shoots by protecting the assembly of photosystems, enhancing the biosynthesis of sucrose and inhibiting the degradation. Under salt stress, the genes encoding photosynthetic proteins were also shown to present different expression regulations in distinct sweet sorghum genotypes. The genes encoding *Lhca1* and *Lhcb1-5* were downregulated in both salt-tolerant and salt-sensitive sweet sorghum lines under salt stress [98]. As a C4 plant, sweet sorghum uses NADP-malic enzyme, ribulose-bisphosphate carboxylase, phosphoenolpyruvate carboxylase, and pyruvate orthophosphate dikinase as the key enzymes in the dark reaction of photosynthesis. When treated with NaCl, the genes encoding these enzymes in sweet sorghum were downregulated, indicating that salt stress reduced CO_2_ assimilation. However, genes encoding other enzymes involved in carbon fixation in photosynthetic organisms, such as NADP+-malate dehydrogenase, were extremely enhanced by salt stress in only salt-tolerant sweet sorghum genotypes. This could increase the levels of CO_2_, pyruvate, and *NADPH*, which enhance CO_2_ assimilation. So far, the molecular mechanism of comprehensive salt responses in sweet sorghum has been largely unknown. It is necessary to draw great attention to the mechanism of salt tolerance and the functions of salt-tolerant genes/proteins in sorghum.

In order to study the photosynthetic system of sweet sorghum under salt stress in depth, we also need to keep digging into the gene editing system of sorghum and the role of phytohormones on the photosynthetic system of sorghum. Currently, GAs have been shown to promote Na^+^/K^+^, nutrient and ROS homeostasis, alleviate damage to the PS II system from salt stress, and can protect the normal processing function of the PS II reaction center and maintain normal electron transfer, thus maintaining high photochemical conversion efficiency to enhance plant tolerance to salinity [99]. The regulation of sorghum by GAs and their interactions has become one of the important mediators of salt-tolerant crops.

#### 6.1.3. Related Pathways of Osmotic Regulation

Metabolic imbalances caused by salt stress are almost always associated with increased production and accumulation of ROS, which in turn cause oxidative stress and damage membrane lipids, DNA, and proteins. A transcriptome study of two different salt-tolerant of Ceanothus under salt stress revealed numerous genes are involved in pathways such as POD metabolism and glutathione metabolism for the synthesis of ascorbic acid and other substances after salt stress, and most genes showed upregulation [100]. A total of 69 *DEGs* related to the ROS scavenging system have been identified in sorghum seedlings after salt stress, and most genes are upregulated, suggesting that sorghum is better able to scavenge the reactive oxygen system and enhance salt tolerance by enhancing this system [43]. To explore the expression of key genes of the antioxidant system in response to high salt stress, Lacerda et al. [101,102] sequenced sorghum roots and leaves and identified seven classes of ROS-scavenging genes, including SOD, POD, and CAT, among differentially expressed genes [103]. Based on GO and Kyoto Encyclopedia of Genes and Genomes (KEGG) analysis of the differentially expressed genes, the ROS scavenging genes involved were mined and the transcriptome sequencing results were validated using qRT-PCR. The results showed that [104] 7239 genes had more than a 2-fold change in gene expression in sorghum after salt treatment, including 4037 upregulated genes and 3162 downregulated genes that were significantly enriched in 12 KEGG metabolic pathways. The expression of 45 genes related to ROS scavenging was significantly altered under salt stress, including 28 upregulated genes and 17 downregulated genes. The results suggest that ROS scavenging is an important mechanism for sorghum to resist salt stress [105].

In summary, the antioxidant mechanism in plants under high salt stress is mainly the upregulation of antioxidant gene expression to initiate the corresponding physiological responses, enhance the scavenging of ROS produced by salt stress, alleviate osmotic stress, and thus resist the adverse environment.

#### 6.1.4. Effect on Membrane Lipid Metabolism in Sorghum Seedling Leaves under Salt Stress

Salt stress can cause membrane lipid changes (including in membrane lipid content and signal lipid activity) and ultimately change the biological characteristics of membrane lipids [81]. Changes in plant membrane lipids cause lipid biosynthesis and metabolic enzyme regulation [106]. A Kyoto Encyclopedia of Genes and Genomes (KEGG) analysis was also performed on the *DEGs* to explore metabolic pathways in the transcriptome of sweet sorghum [107].

In studies with sorghum, 25 genes were found to be localized in two pathways related to membrane lipid metabolism, 11 of which were localized in glycerolipid metabolism and the other 14 in glycerophospholipid metabolism. In a comparison of membrane lipid changes under salt stress in salt-tolerant sorghum self-incompatible lines, and salt-sensitive materials, either glycerolipid metabolites or glycerophospholipid metabolites were identified as being involved [108].

Glycerolipids are the most abundant lipids in higher plants. They include phospholipids, glycolipids, oils, and extracellular lipids, which are widely involved in different biological processes.

It can be seen from the KEGG pathway of glycerophospholipid metabolism that phosphatidylcholine (PC) is an important intermediate phospholipid and is the main component of the membrane. Lipid metabolism has been found to contribute significantly to the ability of plants to survive under saline stresses [109]. The addition of choline, a key substrate for PC biosynthesis, resulted in enhanced salt resistance in wheat, which was evidence of the positive regulatory role of PC [110]. We found that *DEGs* encoding phospholipase A1 were significantly downregulated in sorghum. Increased PC accumulation appears to be an indispensable adaptive response of plants to salt stress.

Expression of *SORBI_3003G360700*, encoding GPAT, is upregulated in salt-tolerant materials during lipid metabolism in sorghum, where overexpression of *SsGPAT* in Arabidopsis prevents the reduction of chlorophyll content and helps maintain unsaturated fatty acid levels. In summary, the adjustment of lipid metabolism in response to salt stress could contribute to an increase in salt tolerance, as displayed by the improved expression profile of GPAT, coupled with TAG mobilization performed cooperatively in mediating salt-defensive responses in sweet sorghum leaves. This provides new insight into the potential role of the membrane lipid regulatory network in the salt tolerance mechanism in sweet sorghum.

### 6.2. Application of DNA Methylation Modification under Salt Stress in Sorghum

**Figure 5:**
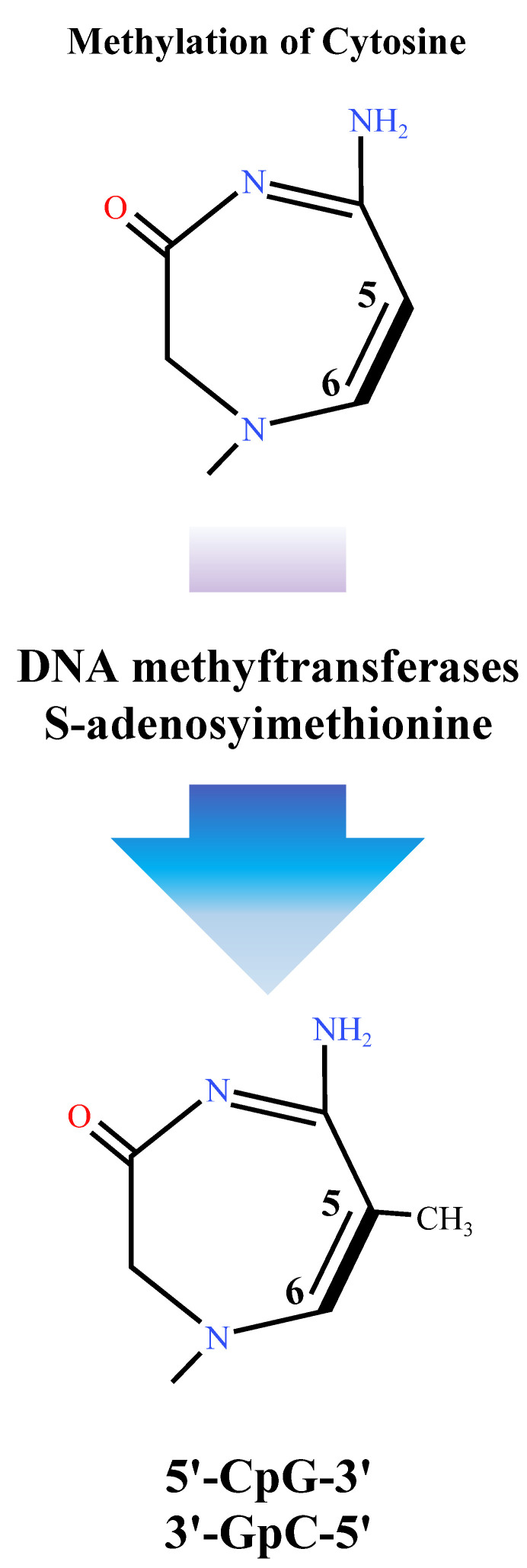
DNA methylation.

Epigenetics refers to changes in gene expression levels based on nongenetic sequence alterations, such as DNA methylation, histone modifications, and chromatin conformational changes. Of these, DNA methylation is an important modification in epigenetics, which generally refers to the change in cytosine at the CpG site to 5-methyl cytosine in genes or genomic sequences under the action of methylation transferase cytosine (Figure 4) [111]. Methylation in plants usually occurs in three cytosine forms, such as CG, CHG, and CHH (H for A, T, or C), with CG modifications as the major form (Figure 5) [112]. In addition, DNA methylation is characterized by species- and tissue-specific and developmental differences, with differences in methylation modifications across species, tissues of the same species, or developmental stages [113,114].

DNA methylation, as one of the important epigenetic mechanisms, plays an important role in life activities. In addition, methylation has been proven to be part of the defense system in prokaryotes. However, recent studies revealed that methylation performed different functions in eukaryotes, such as in genome defense by silencing transposable elements or by maintaining transgenerational genome integrity [115]. Recently, DNA methylation provided heritable epigenetic markers of the cellular functions of plants by directing the developmental program of an organism to stabilize gene silencing [116]. Current research on the regulatory mechanisms of DNA methylation in plants is focused on adverse stresses such as low temperature, drought, and salt stress, which can regulate (repress or activate) gene expression by altering DNA methylation modifications.

Plants live in a constantly changing environment and therefore have to develop necessary responses and defense mechanisms. DNA methylation modifications have been found to play an important role in abiotic stresses [117,118]. Through dynamic changes in their own DNA methylation levels and patterns, plants regulate the expression of stress-responsive genes, thereby enhancing their resistance to stress. Salt stress is a well-studied stress that causes changes in DNA methylation levels in plants. Zhong and Wang examined the changes in 5-methylcytosine content in the DNA of wheat leaves and roots after salt stress and found a significant decrease in the content of 5-methylcytosine in the DNA of wheat leaves and roots after NaCl treatment [119]. It can be inferred that DNA methylation occurs in plants under salt stress [120]. Li et al. found that the DNA methylation rate of the root genome is lower than that of the control after treating it with different concentrations of NaCl [121]. Marconi et al. [122] applied the MSAP technique to analyze changes in DNA methylation in two varieties of kale-type oilseed rape, salt-resistant and salt-sensitive, under salt stress and found that only a few DNA fragments of the oilseed rape seed genome underwent methylation variation. Salt stress induced the onset of demethylation and hypermethylation. Twenty-four differential fragments with altered methylation patterns were recovered and cloned for sequencing. The sequencing results were analyzed by BLAST, and we found that the differential fragments were highly homologous to the resistance genes of *Arabidopsis thaliana*. Choi et al. showed that the tobacco gene *NtGPDL* could be induced by abiotic stresses (heavy metals, low temperature, and salt) but not by biotic stresses. Similarly, salt stress treatment of the salt-tolerant plant ice-leaved *Hinaka* flower indicated that CCWGG sequences were also hypermethylated under salt stress conditions, suggesting that new epigenetic variation generated in salt stress may contribute to plant adaptability and phenotypic diversity [123]. Wang et al. also used the MSAP (methylation-sensitive amplification polymorphism) technique to identify the salt-tolerant rice variety FL478 and the salt-sensitive rice variety IR29 under salt stress and found that the DNA methylation levels in the root systems of both rice varieties decreased compared with the control, and the salt-sensitive rice variety IR29 was more significant. The level of DNA methylation in the root system of both rice varieties were reduced compared with the control, and the salt-sensitive rice variety IR29 was more significant [124]. Li et al. analyzed the response of red flax seedlings to salt stress using 5-ANZAC treatment and found that compared to the control group, the antioxidant enzyme activity was significantly increased, and the ROS content was reduced in the treated group [125]. The MSAP results also showed that red flax seedlings responded to stress by significantly reducing the level of DNA methylation and thus regulating the expression of stress-responsive genes. Thus, as a type of epigenetic modification, DNA methylation modifications play an important role in plant stress response. Dynamic changes in DNA methylation levels and the expression of stress-responsive genes, in turn, confer adaptability to abiotic stresses.

#### Functional Analysis of Sorghum Genomic DNA Methylation Differential Fragment Clones Related to Sequence

By sequencing 54 differentially expressed loci screened in sorghum, glycosylation modifications are used to maintain hormone homeostasis in plants and to regulate the synthesis and storage of secondary metabolites by altering the bioactivity, solubility, and translocation of receptor molecules within the cell and organism. Catalytic glycosylation modifications cannot be achieved without the action of uridine diphosphate glycosyltransferase (UGT), which catalyzes glycosylation reactions by transferring already activated glycosyl groups to small plant compounds. This glycosylation of small molecules is the mechanism by which plants maintain their metabolic homeostasis in response to stress. The glycosyltransferase gene *Twi1* plays a role in signaling and enables plants to respond rapidly to leaf injury and biotic stress. Recent studies have shown that the glycosyltransferase gene *UGT85A5* was transferred into tobacco seeds and subjected to salt stress. Observations and measurements of physiological and biochemical parameters compared with wild-type tobacco revealed for the first time that the glycosyltransferase gene *UGT85A5* plays an important role in enhancing the resistance mechanism of salt tolerance in plants. The expression of the wheat glycosyltransferase genes *TaUGT1* and *TaUGT2* was analyzed by real-time PCR, and it was found that these two glycosyltransferase genes were inhibited and induced by *E. ramorum*, respectively, under salt stress, which may be related to the resistance mechanism of wheat to salt stress.

The leucine sequence receptor kinases (LRR-RLKs) isolated in *Arabidopsis thaliana* are one of the largest families of proteins that play key roles in signaling pathways in animals and plants, regulating growth, development, differentiation, cell death, and defense responses to pathogenic bacteria. Neuronal manipulator proteins (MAMLs) genes encode key activators of signaling pathways. The transcription factor CSL of DNA and activated signal-receiving receptors are manipulated by MAMLs to form a functional complex that regulates the cellular signaling pathway and is important for the transcription process.

We have discussed the understanding of genetic and epigenetic regulation of the molecular response of sorghum to salt stress. They cascade response signals responsible for modifying host epigenetic levels, i.e., histone modifications (histone acetylation/deacetylation), DNA methylation (DMT and DDM) and chromatin tin remodeling. All these changes governed by the epigenetic processes help the stressed plant to keep an eye on the equilibrium of plant growth and development along with the defense in sorghum [126]. To date, we do not fully understand the genetic and epimagnetic mechanisms of action under salt stress in sorghum [127]. Utilization of genome-editing enzymes such as transcription activator-like effector nucleases (TALENs) and CRISPR-Cas9 system can aptly be applied to edit the genome of the stress-subjected sorghum to edit and know the exact function of the particularness expressing exponentially during the environmental stresses [128,129].

Thus far, not much research has been performed on sorghum DNA methylation. However, sorghum, like other crops in the grass family, contains a large number of repetitive sequences. In addition, scientists completed the sequencing of the sorghum genome using whole-genome bird shot sequencing in 2007, which showed that the sorghum genome has about 10,000,000 genes (more than *rice*), with a billion nucleotides. These regions of complex repetitiveness make up a large proportion of both the rice and sorghum genomes and suggest that they share a common overall genome structure as *Graminaceous* plants. Comparative genomic studies have shown that, although sorghum and maize, as well as wheat, barley, rye, maize, and rice, have different genome sizes and chromosome numbers, the results of comparative mapping show that their genomes are highly conserved, with widespread chromosomal co-linearity and intergenic homology.

Figure 5 DNA methylation involves a covalent addition of a methyl group (-CH3) to the 5’carbon of the pyrimidine ring of deoxycytidine (dC), which leads to the creation of 5- deoxymethylcytidine (dmC). The reaction is catalyzed by DNA methyltransferases, whose substrates are palindromic DNA sequences 5′-CG-3′, which are also referred to as CpG dinucleotides [111].

**Figure 6:**
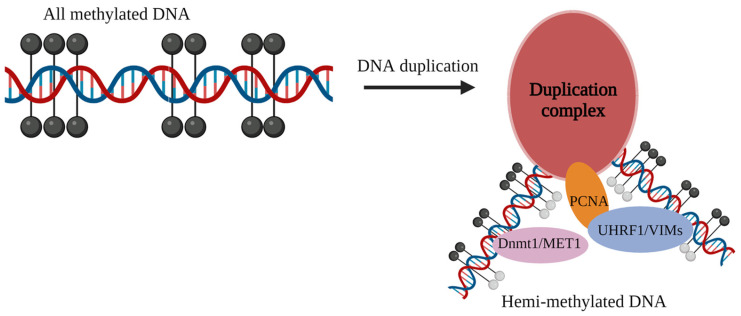
Cytosine DNA methylation.

Figure 6 Cytosine DNA methylation is a stable epigenetic mark that is critical for diverse biological processes, including gene and transposon silencing, imprinting, and X chromosome inactivation. Our discovery and understanding of the pathways used to accurately target, maintain and modify DNA methylation patterns reveal unexpected mechanistic similarities between these organisms. Key roles for small RNAs, proteins with methylated DNA binding domains and DNA glycosylases in these processes have emerged. Drawing on insights from both plants and animals should deepen our understanding of the regulation and biological significance of DNA methylation [130].

### 6.3. Application of Long Noncoding RNAs (lncRNAs) in Sorghum Research under Salt Stress

A growing number of studies show that lncRNAs exert regulatory effects on gene expression levels in the form of RNAs. Taking advantage of second-generation sequencing technologies and bioinformatics approaches, many lncRNAs have been predicted or identified in plants, such as *Arabidopsis* [131,132], wheat [133], maize [134,135], and rice [136], to play important roles in various biological processes in plant development and stress response. Related studies have demonstrated that lncRNAs respond to abiotic stresses, including drought stress, salt stress, and low-temperature stress.

Plants respond to stress by undergoing different changes in their metabolism, physiology, and growth in response to adversity. Plant lncRNAs act as regulators during stress, affecting gene expression by mimicking targets and interfering with transcription and DNA methylation [137]. High-throughput sequencing revealed that a large number of lncRNAs responding to osmotic and salt stress were present in alfalfa, and they acted in concert with protein-coding genes to regulate adversity stress [138]. In a study exploring salt stress in cotton, Zhang et al. [139] found that lncRNA973 enhanced salt stress tolerance in cotton, while downregulation of lncRNA973 expression reduced salt tolerance in cotton and affected the expression of some genes associated with salt stress. This provides new clues to elucidate the response mechanisms of salt damage in cotton. lncRNAs in rice salt stress have been found to be involved in post-transcriptional regulation by polyadenylated lncRNAs, which are co-expressed with protein-coding genes in the regulation of some stress responses and thus play an important role in rice growth [140].

However, little is known about how lncRNAs in sweet sorghum respond to salt stress. Through previous research, we identified 126 and 133 differentially expressed lncRNAs in the salt-tolerant M-81E and the salt-sensitive Roma strains, respectively. Salt-stress-induced three new lncRNAs in M-81E and inhibited two new lncRNAs in Roma. These lncRNAs included *lncRNA13472*, *lncRNA11310*, *lncRNA2846*, *lncRNA26929*, and *lncRNA14798*, which potentially function as competitive endogenous RNAs (ceRNAs) that influence plant responses to salt stress by regulating the expression of target genes related to ion transport, protein modification, transcriptional regulation, and material synthesis and transport. Additionally, M-81E had a more complex ceRNA network than Roma.

Under salt stress conditions, the expression levels of sorghum *SORBI_3009G208000* and *SORBI_3002G237000* were downregulated, while the expression of the corresponding lncRNAs and miRNAs was upregulated. We speculate that salt-induced damage may exacerbate the degradation of target genes miRNAs and that plants respond to salt stress by upregulating lncRNA expression to reduce mRNA degradation. In addition, *SORBI_3002G237000* encodes bundle protein-like arabinogalactan protein 1 (FLA1), which is involved in salt stress response [141]. According to the study, the expression levels of most wheat genes encoding FLAs were found to be downregulated under abiotic stress, which is consistent with our findings. The exact role of these two genes in sweet sorghum under salt stress remains to be investigated [142].

With the rapid development of high-throughput sequencing technology, more and more lncRNAs have been discovered in plants. In recent years, the research on plant lncRNAs has also gradually accelerated. It has been confirmed that plant lncRNAs are involved in plants’ biological processes, such as flower-forming transition, pollen development, and adaptation to stress. However, compared with mammals, there is less information on the transcriptional regulation and molecular evolutionary mechanisms of lncRNAs involved in plant growth and development. By combing through the plant lncRNAs studied in recent years, we can see that plants have certain similarities with animals in terms of structure, origin, and molecular function and show some unique regularities. There is still much room for research on plant lncRNAs. Future research on the involvement of lncRNAs in regulating the mechanism of adversity can be carried out in the following three aspects:

(1) Using modern high-throughput sequencing technology based on the whole genome, large-scale mining of plant adversity-related lncRNAs from the transcriptome level.
(2) Using bioinformatics analysis technology for bootstrap gene prediction, with mRNA co-expression analysis and mock miRNA endogenous pseudo-target gene identification, to provide a basis for in-depth functional identification of lncRNAs.
(3) Although a large number of studies have identified lncRNAs involved in biotic and abiotic stresses in crops, research investigations in sorghum, peanut, and oats are still limited, and the exact genome annotation and functional significance of many lncRNAs are still not known. New sources of lncRNAs are still being discovered, and methods for their classification are still being updated. At the same time, lncRNAs do not function individually, often interacting with other noncoding and protein-coding genes to perform their functions. It is not yet clear how lncRNAs interact with other noncoding RNAs, such as circRNAs in plants. If we want to further explore the regulatory mechanisms of lncRNAs in crops, we need to combine several disciplines, such as bioinformatics, genetics, etc. Meanwhile, with the further development of related technologies, we will have a more complete understanding of the functions and mechanisms of lncRNAs, and lncRNAs will play a more important role in crop genetic breeding, biological resources development, plant cell engineering, and other fields. They will also play a more important role in crop genetic breeding, biological resource development, and plant cell engineering.

Therefore, lncRNAs in plants can be considered essential elements of gene regulation. Future research on lncRNAs will reveal their more complete functions, improve agricultural production, and solve many mysteries in plants.

### 6.4. Application of the Metabolome in Sorghum Research under Salt Stress

There are approximately 200,000 metabolites in plants, including primary metabolites such as proteins, sugars, and lipids and secondary metabolites such as phenols [143]. Metabolites are the end products of cellular regulatory processes at levels that can be considered the ultimate response of a biological system to genetic or environmental changes. Metabolomics is a modern instrumental analysis with high throughput, sensitivity, and resolution, combined with chemometric methods such as biostatistical data processing and pattern recognition, which can provide clear information on plant phenotypes under specific environmental conditions. Changes at the upstream gene and protein level can be amplified in the many metabolites downstream and make these changes more observable, thus better explaining the interaction between biological systems. The method provides clear information on plant phenotypes under specific environmental conditions [144].

Plants can alleviate osmotic stress, remove excess ROS, and prevent damage to their cell membranes by regulating the number and type of metabolites (mainly small molecules such as amino acids, soluble sugars, and organic acids) in their metabolic processes [145,146,147].

#### Analysis of Flavonoid Genes and Related Metabolites

The plant response to salt stress is multilayered and complex. Quantitative and qualitative analysis of metabolites (types of different metabolites, changes in levels, and major metabolic pathways) in salt-stressed sorghum seedlings using high-throughput metabolomics techniques can help us to unravel the metabolic mechanisms of salt-stressed sorghum [148]. Gene–metabolite correlation networks can be used to elucidate functional relationships and identify new regulatory factors. These results suggest that saline stress changes the expression of genes involved in flavonoid biosynthesis, thereby affecting the biosynthesis of flavonoids and derivatives in sorghum under salt stress. Flavonoids are a class of plant secondary metabolites. Flavonoids act not only as scavengers of ROS in plants but also as signal molecules to regulate biological processes [149]. Flavonoids can regulate the expression of related genes by interacting with transcription factors, kinases, histones, ABC transporters, aminopeptidases, and other targets. A common differential metabolic pathway, namely the flavonoid biosynthesis pathway, was found by comparing salt-stress-treated samples [150]. It has been reported that the regulation of flavonoid biosynthesis is controlled by *MYB* transcriptional factors [151], which are largely conserved in plants [152]. The *MYB* transcription factors (TFs) were identified, and expression levels were analyzed. Heatmaps showed that *MYB* TF expression levels were significantly increased under both medium and heavy salt. Thus, *MYB* TF may modulate flavonoid biosynthesis. *MYB* TF of different groups may play different roles in flavonoid biosynthesis [153]. Studies have found that *AtMYB11*, *AtMYB12*, and *AtMYB111* in *Arabidopsis* can independently activate genes related to flavonoid synthesis. In grapes, both *VvMYB5a* and *VvMYBF1* are identified as activators that regulate flavonoid synthesis. When sorghum plants are subjected to salt stress, the expression of the flavonoid gene is significantly increased, and the content of flavonoids in leaves is increased. It is believed that overexpression of the *MYB* gene in sorghum can enhance the tolerance of sorghum plants to salt stress [154].

Using a metabolomic analysis, we could identify the most prominent metabolites in different organs of sorghum, their changes during plant growth, and the diversity of plant metabolomes under different crop conditions. Dhurrin is one of the most peculiar secondary metabolites produced by sorghum. Dhurrin is a cyanogenic glycoside, a class of metabolites that, upon tissue disruption, are hydrolyzed by endogenous β-glucosidases into cyanohydrin aglycone, which in turn releases the toxic hydrogen cyanide (HCN) [155]. In studies, dhurrin was found to be particularly abundant at Ss [156]. On the other hand, dhurrin, like other cyanogenic glycosides, may serve additional functions, such as resistance against abiotic stresses and nitrogen storage/buffer [157,158].

Metabolomics has expanded the understanding of the molecular mechanisms of plant tolerance to abiotic stresses and can detect most of the metabolites in response to stress. There are already studies on the identification of differential metabolites by metabolomics and their action on plants by exogenous application in the form of adversity, such as malonic-acid-soaked wheat seeds or foliar spraying of exogenous 5-aminolevulinic acid in alfalfa. These biomarkers can improve drought tolerance, exogenous application of alginate to periwinkle can promote antagonism to low-temperature stress, and exogenous melatonin can alleviate the inhibitory effect of salt stress on cotton seeds. In contrast, most of the studies on sorghum have been limited to the identification and screening of differential metabolites, so the next step could be to validate the screened functional metabolites by exogenous incorporation and analyze the mechanism of action of exogenous metabolites through changes in physiological and biochemical properties and transcript levels to assist with breeding salt-tolerant varieties.

## 7. Conclusions

Several abiotic stresses, including salt stress, frequently hinder plant morpho-physiological, biochemical, and molecular activity. In this review, the exogenous application of GA can mitigate the negative effects of salt stress on sorghum germination and seedling growth to a certain extent.

## 8. Future Research Trends

Taken together, the above analysis reveals that the effect of salt stress on plants is complex, and there are still many issues involved that current research cannot yet explain [16,159]. Saline soils are a great challenge for today’s society. Soil remediation using sorghum is a green, economical, and sustainable technology with great potential value. Adding GAs is a major direction for future ecological development, both in terms of ecological and economic benefits. It is conducive to improving the safe utilization of herbaceous plants and can be an important target for research on saline land restoration [160]. Therefore, future research and discussion of the improving saline land environment from GAs can be conducted from the following aspects. Firstly, the research on the effect of exogenous hormones on crops usually focuses on the germination and seedling stages. More studies are needed to study the effects of GAs as the whole reproductive period of crops is not well studied. Secondly, exogenous GAs are not widely applied in crop production due to their high cost, ease of aqueous solutions, and difficulty controlling concentrations. More studies are needed to solve these problems and use them at suitable levels to promote crop plant growth under salt stress. Thirdly, the research on GAs in the field of crop stress resistance is mostly on single stress factors and less on multifactor coupling. However, in actual crop production, multiple stresses usually occur simultaneously, such as high temperature and drought, heavy metal pollution and disease, low temperature, salt stress, etc. Therefore, more studies should be performed that consider multiple stresses at once. Additionally, this review gives valuable insight into the phenotypic and physio-biochemical behaviors of contrasting sorghum genotypes, making it possible to decipher the genetics underpinning salt tolerance. These findings potentially pave the way for improving salt tolerance by exploiting suitable candidate genes and associated regulatory networks to develop effective genome-assisted breeding strategies for the genetic improvement of sorghum.

## Data Availability

Not applicable.

## Abbreviations

GA_3_	gibberellic acid
ROS	reactive oxygen species
SOD	superoxide dismutase
POD	peroxidase
CAT	catalase
ATP	adenosine triphosphate
GO	Gene Ontology
IAA	Indole-3-aceticacid
ABA	abscisic acid
BR	Brassinosteroid
JA	jasmonic acid
DEGs	differentially expressed genes
KEGG	Kyoto Encyclopedia of Genes and Genomes
